# Quality of life after cancer—How the extent of impairment is influenced by patient characteristics

**DOI:** 10.1186/s12885-016-2822-z

**Published:** 2016-10-10

**Authors:** Elisabeth Peters, Laura Mendoza Schulz, Monika Reuss-Borst

**Affiliations:** 1 Clinic for Oncology and Rheumatology, Kurhausstr. 9, 97688 Bad Kissingen, Germany; 2 Clinic for Psychiatry und Psychotherapy, University of Goettingen, von-Siebold-Straße 5, 37075 Goettingen, Germany; 3 Center for Rehabilitation and Prevention Medicine, Frankenstr. 36, 97708 Bad Bocklet, Germany

**Keywords:** Oncological rehabilitation, Quality of life

## Abstract

**Background:**

Although this effect is well known, tailored treatment methods have not yet been broadly adopted. The aim of this study was to identify those patient characteristics that most influence the impairment of quality of life and thus to identify those patients who need and can benefit most from specific intervention treatment.

**Methods:**

1879 cancer patients were given the EORTC QLQ C-30 questionnaire at the beginning and end of their inpatient rehabilitation. Patients’ scores were compared to those of 2081 healthy adults (Schwarz and Hinz, Eur J Cancer 37:1345–1351, 2001). Furthermore, differences in quality of life corresponding to sex, age, tumor site, TNM stage, interval between diagnosis and rehabilitation, and therapy method were examined.

**Results:**

Compared to the healthy population, the study group showed a decreased quality of life in all analyzed domains. This difference diminished with increasing age. Women reported a lower quality of life then men in general. Patients with prostate cancer showed the least impairment in several domains. Patients having undergone chemotherapy as well as radiotherapy were impaired the most. Surprisingly, TNM stage and interval between diagnosis and rehabilitation did not significantly influence quality of life. Global quality of life and all functional domains significantly improved after a 3-week rehabilitation program.

**Conclusions:**

Despite an individualized and increasingly better tolerable therapy, the quality of life of cancer patients is still considerably impaired. However, systematic screening of psychosocial aspects of cancer, e.g. quality of life, could enable improved intervention.

## Background

The term “Health Related Quality Of Life” (HRQOL) describes the influence of a person's health status as reflected in his quality of life. Oncological patients often report a strong impairment of HRQOL compared to the healthy population [1, 2]. Numerous possible causes have been discussed including the following:

1. Although survival rates are on the rise and cancer is increasingly developing from a lethal to a chronic disease [3], it is still the most dreaded disease in Germany [4].
2. Information about current and increasingly better tolerable treatment modalities is not very widespread.
3. The mental coping with a cancer diagnosis accompanied by fear, helplessness and despair is a major challenge for most patients.
4. Cancer related fatigue (CRF) is the most commonly reported symptom [5–9]. CRF is experienced as a physical, cognitive or emotional exhaustion, much greater than the normal level of every-day exhaustion and which is not improved by recreation periods alone [8, 9]. Multiple domains of HRQOL are affected by CRF, especially patients’ physical and emotional functioning [5, 7, 8].
5. Many patients report a distinct change in their social environment during their illness. Not only do others withdraw from patients, out of insecurity as to how to interact with the patient or the fear of not being able to cope, but patients themselves withdraw from others because they also feel insecure or do not want to burden others with their troubles. As a result, valuable social impulses for the patients become rare.
6. Last but not least, in many cases the enduring period of disease will pose a financial challenge to the patients when costs rise due to additional contributions to medical treatment, external household help, etc. while incomes decline due to sickness leave and retirement payments being lower than previous earnings.

Since 1980, many scientific papers concerning the various domains of HRQOL in tumor patients have been published worldwide [10]. For several HRQOL domains and symptom items correlations with patients’ survival rates have been found [5, 11–14]. As a consequence, the investigation of HRQOL has received the same level of importance as biometric data and clinical parameters in many studies. However, HRQOL remains impaired in affected patients. The aim of the current study was to detect certain features of patients that influence the severity of impairment of HRQOL in a large cohort of German cancer patients. Interesting factors were age, sex, tumor site, TNM stage, treatment method, and time interval between diagnosis and study participation. Thus, hopefully the results can support the development and broad implementation of intervention programs especially for those patients who need it the most.

## Methods

### Procedure

Between September, 2007 and May, 2011 all oncological patients admitted to the rehabilitation clinic were given the EORTC-QLQ C-30 [15] to complete at the beginning and end of their inpatient rehabilitation. During their initial examination with the doctor in charge, they were informed about the use of their questionnaires. The only exclusion criterion was insufficient skills of the German language.

### Sample

**Fig. 1:**
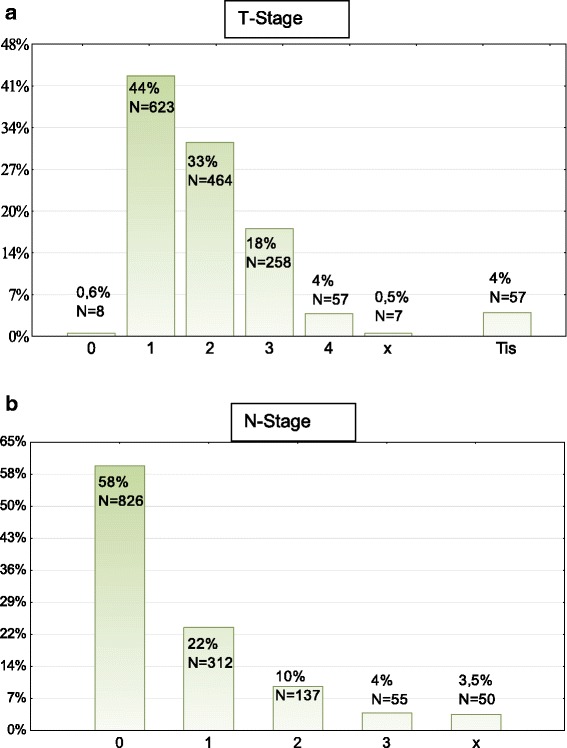
Depicts the distribution of T stages (**a**) and N stages (**b**) in the study population

*N* = 1879 patients were recruited for the study. They had a mean age of 57.03 years (SD = 11.41) and were mostly female (71 %). The time interval between first diagnosis and rehabilitation averaged 16.3 months. The most common diagnosis was breast cancer (45 %), followed by leukemia/lymphomas (13 %), colorectal cancer (12 %) and malignancies of the female genital organs (9 %). 31 % of the patients had received both chemotherapy and radiotherapy. 23 % had only received chemotherapy and 24 % had only received radiotherapy. The T and N stage distribution are depicted in Fig. 1. Only 9 % of all patients had metastatic disease.

### Measures

The EORTC QLQ C30 is presently the most frequently used questionnaire on this topic in Europe [9]. It divides health related quality of life into five functional domains (physical functioning, role functioning, emotional functioning, cognitive functioning, and social functioning), three symptom domains (fatigue, nausea and vomiting, pain), six further additional cancer associated symptoms (dyspnea, appetite loss, insomnia, constipation, diarrhea, financial difficulties) and gives a global value for quality of life. Thereby as well as in the functioning scales, high values represent a high quality of life. For symptoms and symptom scales, high values represent high symptomatology. For the current study, only global quality of life, the functioning scales and the fatigue scale were evaluated (see Table 1).

**Table 1:** EORTC-QLQ-C30 questionnaire: evaluation of gQoL, functioning scales and fatigue

− − − − − − −	Global quality of life (gQoL) Physical functioning (PF) Emotional functioning (EF) Cognitive functioning (CF) Role functioning (RF) Social functioning (SF) Fatigue

For interpretation of QLQ C-30 data, reference data from the German healthy population were used [16]. Additionally, score differences between groups or different time points were analyzed using a classification of Osoba et al. [17]: From one of their studies it became apparent that patients experience a difference in scale values of 5 to 10 points as “a little”, 10 to 20 points as “moderate”, and ≥20 as “very much”. Therefore, differences of less than 5 points are not clinically relevant.

### Statistical analyses

All questionnaire data were transferred to a Microsoft Excel table and scored in accordance with the official EORTC QLQ C-30 manual [15]. With statistical programs STATISTICA (version 9.1 and 10.0) and the free programming language R [18] the higher analyses were carried out. Two independent samples were compared using the Wilcoxon-Mann-Whitney test, multiple comparisons were done with the R package “nparcomp”. Level of significance was set to α = .05. In this paper, only those group differences are described which are statistically and clinically meaningful.

## Results

### Quality of life in patients and healthy controls

The scale values for global QoL, the six functioning scales and the symptom scale „fatigue” from the study sample were compared to a sample from the healthy German population. These randomly selected 2041 German adults were chosen by using the random-route technique (random selection of street, house, flat and target subject in the household) and provided information about their quality of life. According to the authors the sociodemographic data were representative of the adult German population [16]. For each scale, a clinically meaningful difference was found which can be labeled as “strong” according to Osoba [17]. According to his research a difference of 5–10 points on a 0–100 point scale has to be considered as minimally clinically important. Differences between 10 and 20 points have a moderate clinical meaning, and >20 points have a strong clinical meaning. Compared to the healthy population, global QoL and functioning were remarkably reduced and fatigue was increased in the study population (see Table 2).

**Table 2:** Scale mean scores (M) and standard deviations (SD) of the study population compared to a healthy sample

Score	M ± SD Study population	M ± SD Healthy population ^a^	Difference
gQoL	49.2 ± 22.0	70.8 ± 22.1	21.6
PF	66.7 ± 20.9	90.1 ± 16.7	23.4
EF	50.4 ± 28.6	78.7 ± 21.0	28.3
CF	65.5 ± 29.4	91.2 ± 17.0	25.7
RF	51.5 ± 30.9	88.0 ± 22.9	36.6
SF	58.4 ± 32.0	91.0 ± 19.4	32.6
F	56.4 ± 27.6	17.0 ± 22.0	39.4

*Abbreviations*: *gQoL* global quality of life, *PF* physical functioning, *EF* emotional functioning, CF cognitive functioning, *RF* role functioning, *SF* social functioning, *F* fatigue

^a^ Schwarz & Hinz, 2001

### Quality of life in different patient groups

Different socioeconomic parameters were analyzed that might affect quality of life in cancer patients; these being Age, Gender, Sex, Tumor Entity, TNM Stage, Previous Treatment, Time Interval since Diagnosis.

- Age:
  A comparison of scale values from participants of different age groups was done. We are only reporting those results that are statistically and clinically significant [17]. Participants younger than 39 years had a slightly higher physical functioning than those older than 60. Emotional functioning was most impaired in the 40–59 year olds, while no difference between the two other groups was found. Social functioning was most impaired in the youngest group, followed by the middle and then by the oldest group. Fatigue symptoms were more pronounced in the middle than in the youngest group (see Table 3). For each of the age groups scale values differed significantly from values of similar age groups in the healthy population. However, the differences were more pronounced in the youngest group (≤39) and lowest in the oldest (≥60) for most scales. These results are shown in Table 4.

**Table 3:** Contains all clinically meaningful differences in scale values between participants in different age groups

Score	Age ^a^	*N*	M ± SD	*p*-value	Difference
PF	≤39	129	71.7 ± 20.8	.000	7.3
	≥60	671	64.5 ± 20.5		
EF	≤39	126	54.47 ± 25.7	.007	7.5
	40–59	1014	46.9 ± 28.9		
	40–59	1014	46.9 ± 28.9	.000	8.2
	≥60	652	55.0 ± 28.0		
SF	≤39	129	52.8 ± 30.1	.001	9.9
	≥60	677	62.8 ± 30.8		
	40–59	1029	56.2 ± 32.8	.000	6.6
	≥60	677	62.8 ± 30.8		
F	≤39	128	51.1 ± 66.7	.047	6.0
	40–59	1039	57.1 ± 77.8		

*Abbreviations*: *PF* physical functioning, *EF* emotional functioning, *SF* social functioning, *F* fatigue

^a^ age (years)

**Table 4:** Shows mean scale values from the study population compared to the healthy population sample [16] in different age groups

Score	Age ^a^	M ± SD study population	M healthy population ^b^	Difference
gQoL	≤39	53.78 ± 20.34	81.70	27.92
	40–59	47.25 ± 22.27	72.85	25.60
	≥60	51.33 ± 21.67	63.55	12.22
PF	≤39	71.68 ± 20.80	98.10	26.42
	40–59	67.52 ± 21.20	94.30	26.78
	≥60	64,50 ± 20,49	82,43	17.93
EF	≤39	54.37 ± 25.71	84.80	30.43
	40–59	46.87 ± 28.85	80.15	33.28
	≥60	55.02 ± 28.02	81.00	25.98
CF	≤39	68.72 ± 26.98	96.95	28.23
	40–59	62.42 ± 30.54	94.30	31.88
	≥60	69.72 ± 27.44	86.30	16.58
SF	≤39	52.84 ± 30.13	97.30	44.46
	40–59	56.19 ± 32.75	92.85	36.66
	≥60	62.75 ± 30.83	86.00	23.25
RF	≤39	54.97 ± 29.83	96.70	41.73
	40-59	50.75 ± 31.15	90.80	40.05
	≥60	51.88 ± 30.76	81.40	29.52
F	≤39	51.13 ± 27.23	7.60	43.53
	40–59	57.12 ± 27.67	12.15	44.97
	≥60	56.51 ± 20.34	23.20	33.31

*Abbreviations*: *gQoL* global quality of life, *PF* physical functioning, *EF* emotional functioning, CF cognitive functioning, *SF* social functioning, *RF* role functioning, *F* fatigue

^a^ in years

^b^ Schwarz & Hinz, 2001; no SD values given
- Gender:
  Between men and women in the study population, clinically meaningful differences were found for physical, emotional, cognitive and role functioning as well as for fatigue. Women reported stronger impairments than men. All found differences were classified as minor according to Osoba [17] (see Table 5).

**Table 5:** Contains all clinically meaningful score differences for men and women

Score	Sex	*N*	M ± SD	*P* value	Difference
PF	Male	533	70.26 ± 21.61	.000	5.01
	Female	1299	65.25 ± 20.57		
EF	Male	528	56.63 ± 28.38	.000	8.88
	Female	1264	47.75 ± 28.30		
CF	Male	534	70.23 ± 27.33	.000	6.60
	Female	1307	63.63 ± 29.99		
RF	Male	524	56.04 ± 31.90	.000	6.52
	Female	1289	49.52 ± 30.31		
F	Male	531	51.04 ± 28.65	.000	7.68
	Female	1290	58.72 ± 26.85		

*Abbreviations*: *PF* physical functioning, *EF* emotional functioning, CF cognitive functioning, *RF* role functioning, *F* fatigue
- Diagnosis:
  Between patients with different diagnoses, statistically and clinically meaningful differences concerning physical, emotional, cognitive, social, and role functioning as well as fatigue were found. Patients with prostate carcinoma reported less impairment than other patient groups in different domains of QoL: physical and role functioning were moderately increased compared to patients with breast cancer and malignancies of the female genital organs; emotional functioning was much higher than for patients with malignancies of thyroid and other endocrine glands; social functioning was moderately increased compared to patients with malignancies of the female genital organs; and fatigue symptoms were less distinct than in patients with breast cancer, malignancies of the female genital organs, with leukemia/lymphomas, and with gastrointestinal carcinoma. Patients with malignancies of thyroid and other endocrine glands reported decreased emotional functioning compared to patients with leukemia/lymphomas, with colorectal cancer, and with gastrointestinal carcinoma. Patients with breast cancer reported slightly decreased emotional and cognitive functioning compared to patients with colorectal carcinoma. Table 6 provides details of these results.

**Table 6:** Depicts statistically and clinically significant differences in scale values between groups of patients with different malignancies

Score	Diagnosis	*N*	M ± SD	*P* value	Difference
PF	Breast cancer	824	66.95 ± 19.58	.035	5.35
	Malignancies of female genital organs	174	61.30 ± 20.26		
PF	Breast cancer	824	66.95 ± 19.58	.033	7.87
	Prostate carcinoma	85	74.82 ± 20.12		
PF	Malignancies of female genital organs	174	61.30 ± 20.26	.000	13.52
	Prostate carcinoma	85	74.82 ± 20.12		
EF	Colorectal carcinoma	211	55.92 ± 28.83	.032	7.09
	Breast carcinoma	801	48.28 ± 28.37		
EF	Colorectal carcinoma	211	55.92 ± 28.83	.002	18.34
	Malignancies of thyroid and other endocrine glands	55	37.58 ± 26.88		
EF	Prostate carcinoma	84	58.73 ± 29.71	.002	21.15
	Malignancies of thyroid and other endocrine glands	55	37.58 ± 26.88		
EF	Hemoblastoses	234	52.03 ± 29.91	.015	14.45
	Malignancies of thyroid and other endocrine glands	55	37.58 ± 26.88		
EF	gastrointestinal carcinoma	93	54.39 ± 29.09	.019	16.81
	Malignancies of thyroid and other endocrine glands	55	37.58 ± 26.88		
CF	Colorectal carcinoma	216	71.45 ± 27.28	.006	8.96
	Breast cancer	826	62.49 ± 30.19		
SF	Malignancies of female genital organs	170	51.57 ± 33.00	.039	14.14
	Prostate carcinoma	87	65.71 ± 29.24		
RF	Breast cancer	820	51.10 ± 29.56	.044	11.35
	Prostate carcinoma	87	62.45 ± 31.15		
RF	Malignancies of female genital organs	168	48.12 ± 31.61	.025	14.33
	Prostate carcinoma	87	62.45 ± 31.15		
F	Breast cancer	822	57.88 ± 26.77	.005	13.31
	Prostate carcinoma	87	44.57 ± 28.73		
F	Malignancies of female genital organs	169	59.17 ± 25.96	.007	14.60
	Prostate carcinoma	87	44.57 ± 28.73		
F	Prostate carcinoma	87	44.57 ± 28.73	.008	14.19
	Hämoblastosen	239	58.76 ± 27.83		
F	Prostate carcinoma	239	44.57 ± 28.73	.017	15.50
	gastrointestinal carcinoma	91	60.07 ± 27.67		

*Abbreviations*: *PF* physical functioning, *EF* emotional functioning, CF cognitive functioning, *SF* social functioning, *RF* role functioning, *F* fatigue
- Previous treatment:
  Patients who had undergone radiotherapy and chemotherapy showed moderately decreased physical functioning compared to patients with other treatment methods (radiotherapy only, chemotherapy only, or other). Other differences were not clinically meaningful (see Table 7).

**Table 7:** Shows the difference in physical functioning between patients with radio- and chemotherapy compared to patients with other treatments

Score	Chemo- and radiotherapy ^a^	*N*	M ± SD	Median	Q25	Q75	*p*- value	Difference
PF	no	1263	67.75 ± 20.90	66.67	53.33	86.67	.001	19.36
	yes	563	48.39 ± 21.08	66.67	46.67	80.00		

*Abbreviation*:*PF* physical functioning

^a^ yes = combination of chemotherapy and radiotherapy, no = any other treatment
- Tumor stage and time interval since diagnosis:
  Comparing different tumor stages, lymph node manifestations, and metastatic spread did not show any significant differences concerning the QoL of patients. Furthermore, there was no correlation with QoL regarding the time interval between diagnosis and rehabilitation.

#### Improvement of quality of life after rehabilitation

All patients included in this study received a comprehensive multi-modal in-patient rehabilitation program of 3-week duration based on a bio-psychosocial and positive health concept which was mainly comprised of psycho-oncological consultations in group and individual sessions, physical activities like Nordic Walking, water gymnastics, physiotherapy and (cardiorespiratory) fitness training as well as vocational therapy, cognitive treatment and support by a social worker if needed. Comparing life quality at the beginning and end of our rehabilitation program gQoL and each functioning scale was significantly improved (*p* < 00,001). Beside global life quality, emotional functioning and role functioning improved the most, i.e. between 10 and 20 points on the 100 point scale (see Table 8).

**Table 8:** Depicts mean quality if life scores before (pre) and after (post) rehabilitation

Rehabillitation	*N*	M ± SD	Median	Q25	Q75	*P*- value	Δ
pre gQoL post gQoL	1850	49,22 ± 22,05	50,00	33,33	66,67	0000	11,84
	1578	61,06 ± 20,16	66,67	50,00	75,00		
pre PF post PF	1832	66,71 ± 20,99	66,67	53,33	80,00	0000	6,70
	1561	73,41 ± 18,97	73,33	60,00	86,67		
prae EF post EF	1792	50,37 ± 28,61	50,00	25,00	75,00	0000	16,29
	1531	66,66 ± 26,70	66,67	50,00	91,67		
prae SF post SF	1835	58,37 ± 32,05	66,67	33,33	83,33	0000	9,07
	1559	67,44 ± 29,54	66,67	50,00	100,00		
prae KF post KF	1841	65,54 ± 29,39	66,67	50,00	83,33	0000	5,35
	1573	70,89 ± 27,66	83,33	50,00	100,00		
prae RF post RF	1813	51,46 ± 30,93	50,00	33,33	66,67	0000	13,69
	1535	65,15 ± 28,59	66,67	50,00	100,00		
prae F post F	1821	56,47 ± 27,61	55,56	33,33	77,78	0,000	8,68
	1553	65,15 ± 26,51	33,33	22,22	66,67		

*Abbreviations*: *gQoL* global quality of life, *PF* physical functioning, *EF* emotional functioning, CF cognitive functioning, *RF* role functioning, *F* fatigue

## Discussion

Having a look at our study population, it becomes clear that the distribution of neither tumor sites nor sex is representative for cancer in general. This is due to a specialization for breast cancer in the study center. Nevertheless, we believe that the registered data are sufficient to deduce some general knowledge about HRQOL in oncological patients.

Patients in the current study scored significantly lower in all domains of HRQOL than the healthy German population. Certainly, the overrepresentation of women in the study group has put a possible bias on the results, because it has often been shown that women regularly report lower quality of life than men on average [16]. This has been confirmed in this study. So perhaps the discrepancy of values between our study group and the healthy population has been slightly overestimated. Nevertheless, the results show a distinct impairment of different areas of life following cancer and treatment. Some patient characteristics can be identified which hint to a particularly high impairment and need of support.

At first sight, regarding the age groups of our study participants, it is difficult to identify a certain trend of age related changes in HRQOL, as there are only minor advantages for different groups in different domains. However, a trend becomes apparent when one compares the age related changes in the study group with those of the healthy population. While in all age groups the study group differs significantly from the healthy population, this difference is by far more pronounced in younger than in older age groups for most of the scales. These results go in line with those of Curt et al. [7] who report that younger tumor patients more often complain about depression, hopelessness and suicidal tendencies than older patients and they feel more limited in social activities. Even the difference in physical functioning diminishes with increasing age although more severe side effects (e.g. heart failure, polyneuropathy) can be expected for chemo- and radiotherapy in older age patients [19, 20]. As differences between the age groups of cancer patients are not very distinct, but a distinct change in the difference between patients and healthy population exists, it has to be assumed that this is due to age-related changes of HRQOL in the healthy population [21]. Maybe in the oldest age group the reference group should not be referred to as “healthy population” as the prevalence of chronic diseases and additional handicaps generally increase in older people, which may then lead to overall decreasing HRQOL. Hence in this group it is not even sure how much HRQOL is specifically impaired by the cancer disease considering the eventuality of other handicaps. On the contrary, members of the youngest and middle age group are compared with people of a life stage that is normally characterized by maximum independency and strength [22]. Here, the discrepancy between the “image of being young, healthy and beautiful” and the actual situation of a cancer patient suffering, for example, from pronounced fatigue after intense treatment, is very obvious. Treatment in younger patients is often intensive, multimodal and lengthy. The diagnosis of cancer affects young patients in a vulnerable phase of their life when their normal private (e.g. partnership) and professional development is interrupted. Thus, healthcare providers should be aware of the symptom burden in this special patient group and regularly monitor HRQOL in young patients to direct attention to psychosocial and vocational problems and ascertain holistic care in this special population.

Concerning tumor site, a trend for patients with prostate carcinoma reporting less impairment in the different domains of HRQOL than patients with other diagnoses becomes apparent. This goes in line with findings of other studies [23]. Why would that be? The general prognosis of prostate carcinoma is comparably positive—but nevertheless, it does not differ much from that of breast cancer [24, 25]. In addition, many men suffer from potency problems after having their prostate carcinoma treated which may correspond to the loss of female identity in women after breast surgery. There may be other reasons why these patients still score higher on the different domains of HRQOL. First, the prognosis of prostate carcinoma is more positive compared to some of the other diagnoses. Second, this specific group of patients consists of men only whereas all other groups contain at least some women. As we already mentioned, women tend to report lower HRQOL than men in general. Third, chemotherapy which (in combination with radiotherapy) causes some of the most severe sequelae in treatment of cancer is rarely applied in prostate carcinoma.

Concerning treatment methods, we found that the combination of chemotherapy and radiotherapy has a remarkably diminishing effect on physical functioning compared to all other treatments while radiotherapy and chemotherapy alone do not. Furthermore, Cancer-related fatigue (CRF) seems to be more pronounced in this treatment regime. It is known that CRF predicts lower physical functioning [26, 27] and that after chemotherapy and radiotherapy 80 % and 90 % suffer from CRF, respectively [5, 8, 28, 29]. Hence, the combination of chemo- and radiotherapy may aggravate CRF and therefore aggravate impairment of physical functioning.

Surprisingly, although one could have expected a lower HRQOL in patients with a more advanced tumor, nodes and metastases stages, these factors did not seem to influence the domains of HRQOL. In some way this corresponds to our clinical perception, which we often experience that patients with early stage cancer and best prognoses are as frightened of progression and tumor recurrence and afraid of death as patients at a later stage. This probably goes back to the previously described fact that cancer is by far the most feared disease in Germany and is mostly thought of as lethal whereas information about developing treatment methods and improving prognoses are not that far spread. Furthermore, the composition of the study group may play a role in this result because most of the participants had low tumor stages (1–2), few affected lymph nodes (stage 0–1) and no metastases at all; so the weight of extremely affected participants in the statistical analyses is very low. Another theory which becomes more and more important in the field of HRQOL and which can be taken for a possible explanation is that of response shift. During the period of (chronic) illness patients gradually change their expectancies and values which can lead to a different concept of HRQOL [30]. Therefore they can still reach similar scores in different HRQOL domains as before but with a different meaning.

From our point of view, perhaps the most important result is that HRQOL does not seem to improve spontaneously over at least the first 16 months on average after diagnosis of cancer. Although most patients in our study have been treated curatively, so that most of them will probably survive their cancer, their life quality remained strongly impaired for many months after diagnosis. This finding is in agreement with other studies [31, 32] and highlights the importance of HRQOL screening and HRQOL specific intervention for oncological patients. Even long-term cancer survivors might profit from rehabilitative intervention. However 46 % of German cancer survivors feel inadequately informed about support offers with their lack of knowledge being mostly associated with older age and lower education [31]. This is particularly important because patients may benefit with regard to their HRQOL from a short intense intervention like a 3 week rehabilitation program as could be shown in our study. Coping and empowerment are key factors within rehabilitation. Substantial components of the rehabilitation are also aerobic physical activities like Nordic Walking, water gymnastics, (cardiorespiratory) fitness training as well as muscle strength training. A positive influence of physical activity on CRF, physical distress, physical functioning and HRQOL has been shown repeatedly [33–35]. Additionally, there are several components which could be classified as belonging to so-called integrative (complementary) medicine: where in regular psycho-oncological sessions and, if needed, individual care can be given. Furthermore, patients can benefit from traditional Chinese medicine (acupuncture), dance therapy or art therapy which can also positively influence HRQOL by helping the patient finding some deeper meaning of his or her illness. The meaning building process is actually discussed as one main factor for successful coping. All those intervention components are not necessarily bound to inpatient rehabilitation but can also be integrated in ambulant care. In addition, screening for psychological and physical impairments will allow tailoring rehabilitative procedures for individual needs.

While oncological treatment methods will develop further to a so called “individualized therapy”, integrative treatment modalities are still somehow neglected. Today, the term “individualized or personalized therapy” stands for therapy based on individual biomarkers. The idea or concept of “individualized therapy” is however broader, reflecting a therapy of individual patient’s needs. The development to a broader understanding of this term and to an anchorage of integrative holistic medicine in oncological care routine has to take place to be able to not only support patients on the somatic but also on the psychosocial level. Monitoring HRQOL can help cancer patients to communicate concerns and symptoms to health care providers that might not be otherwise discussed. Screening for impairments in HRQOL might be a valuable simple tool for detecting those in need of special medical attention and complex rehabilitation interventions. Young female patients having been treated with chemo- and radiotherapy can probably benefit the most regardless of the objective severity of their cancer disease as also has been discussed by Silver et al. [36].

There are some limitations to this study. First, we analyzed patient characteristics separately and not in some kind of multiple regression analysis. Thus, we can only report separate findings although it is likely and in some cases even certain that patient characteristics are interrelated (e.g. diagnosis and sex). Future studies should broaden their approach in this regard. Second, the study is based on a selective subpopulation of patients who underwent inpatient rehabilitation. In Germany, the percentage of oncological patients undergoing inpatient rehabilitation has declined to only 1/3 in the last few years [31]. It is not yet known which factors influence utilization of rehabilitation, but one might speculate that these patients actively seek help from their doctors and are therefore psychologically more strained and/or better informed about rehabilitation programs than others. Thus, generalization of our study results has to be done with caution.

## Conclusion

Persistent and comprehensive impairment of HRQOL in cancer patients on the one hand and the various intervention possibilities on the other hand underline the importance and practicability of a broader consideration of HRQOL as patient reported outcome parameter during and after cancer treatment. Standardized screening instruments like the EORTC QLQ C-30 represent a simple and economic way of screening for physical and psychological impairments and offering appropriate (tailored) intervention programs if needed. The implementation of efficient networks of (ambulant) health-care providers (primary care physicians, clinical oncologists, nurse practitioners, mental health professionals) is urgently needed, in particular since the number of cancer survivors is steadily increasing and many of them have or will have multiple impairments.

## Abbreviations

CRF: Cancer related fatigue
EORTC QLQ C-30: European Organization for Research and Treatment of Cancer, Quality of Life Questionnaire, Core 3.0
HRQOL: Health related quality of life
QoL: Quality of life
SD: Standard deviation
TNM: T: tumor, N: node, M: metastases
